# Postthyroidectomy Throat Pain and Swallowing: Do Proton Pump Inhibitors Make a Difference?

**DOI:** 10.1155/2013/135978

**Published:** 2013-10-09

**Authors:** Faisal Zawawi, Keith Richardson, Rickul Varshney, Jonathan Young, Alex M. Mlynarek, Michael P. Hier, Richard J. Payne

**Affiliations:** ^1^Department of Otolaryngology, Head and Neck Surgery, McGill University, Montreal, QC, Canada H3A 1A1; ^2^Department of Otolaryngology, Head and Neck Surgery, King Abdulaziz University, Jeddah, Saudi Arabia

## Abstract

*Objectives.* Following thyroid surgery patients complain from swallowing disability and throat pain resembling symptoms of laryngopharyngeal reflux (LPR). The purpose of this study is to assess whether proton pump inhibitors (PPIs) given to patients in the first postoperative week relieve the swallowing disability and throat pain complaints. *Materials and Methods.* A questionnaire was given to 523 patients who had thyroid surgery between October 2010 and August 2011. In the first postoperative clinic visit each patient was approached to fill out a questionnaire. 208 patients met criteria, 100 of which were on PPIs (study group) and 108 were not on PPIs (control group). *Results.* When comparing the study group to the control group, the average pain level was 2.57 compared to 3.9 during the first postoperative week, and 1.27 compared to 2.41 at day 7 (*P* value = 0.001). Swallowing disability was also lower in the study group when compared to the control group, 1.87 and 3.12, respectively, during the first postoperative week and 0.87 and 1.76, respectively, at day 7 (*P* value = 0.007). *Conclusion.* Patients treated with PPIs had less pain and swallowing disability in the first week following thyroid surgery, when compared to patients not treated with PPIs.

## 1. Introduction

It has been observed that patients who undergo thyroidectomy complain of throat pain and short-term dysphagia following their surgery [1–3]. Their surgical intervention and traumatic intubation can be explanations for these symptoms. However, no actual cause tends to be found for the majority of cases [1, 2]. These symptoms, that mimic laryngopharyngeal reflux (LPR), cause a relative disability in the short-term period following a thyroid gland surgery [2, 3].

LPR is a common disorder in which patients often complain of throat pain and swallowing discomfort (globus sensation) [4, 5]. LPR can be well controlled by both lifestyle modifications and if needed medications. Proton pump inhibitors (PPIs) are commonly used in the treatment of LPR [4, 5]. Because symptoms of individuals with LPR are similar to those of postthyroidectomy patients, we hypothesized that treating patients with PPIs may be beneficial in diminishing throat pain and dysphagia in patients who have undergone thyroid surgery. 

The purpose of this study is to compare the complaints of throat pain and dysphagia following thyroid surgery in patients given PPIs versus those who were not.

## 2. Material and Methods

This is a prospective case-controlled pilot study conducted at the McGill Thyroid Cancer Centre from October 2010 until August 2011. Ethics approval was acquired through McGill University Institutional Review Board. 

### 2.1. Population and Study Treatment

523 patients underwent thyroid surgery from October 2010 to August 2011; 315 were excluded because they were either already on a PPI prior to surgery or the questionnaire was not filled out properly. The patients also provided demographic information. All patients had thyroid surgery with 1 of 4 surgeons using a similar technique. The study group did not know that the prescribed PPI was addressing their pain and swallowing ability. Further information about the research objective was provided to both groups after filling the questionnaire.

The collected data was then electronically arranged on Microsoft Excel (12.3.1). Statistical analysis was performed on SPSS (20.0.0). The patients were classified into groups: study and control. The study group consisted of the patients placed on PPIs postoperatively. They were instructed to take one pill per day for 14 days. The patients were asked to fill up the questionnaire at their first postoperative visit (suture removal visit), which was scheduled at a similar timeframe for all patients.

### 2.2. Questionnaire

Questions focused on pain and swallowing difficulty during the postoperative period. The patients were asked to classify their discomfort on a numerical pain score scale. There were 8 questions. Three questions were targeting swallowing difficulty, three targeting throat pain (not incision site pain), and two addressing the kind of PPI that was prescribed to the patient postoperatively. The patients were asked to score their pain and swallowing disability in 3 intervals, first postoperative day, mid first week, and at postoperative day 7.

The questionnaire adapted the numerical rating pain scale from 0 to 10 with 10 being the most pain.

The questionnaire was sent to four otolaryngologists independently for face and content validity. Afterwards, five patients and three physicians were recruited to perform the initial pretesting of the questionnaire that generated the third draft. Finally it was sent to McGill University research ethics committee for final validation and approval (Table 1).

## 3. Results

208 patients were included in this study. There were 108 patients in the control group (no PPI) and 100 in the study group (PPI). The mean age = 50.14 (range 21–80), with 84.1% of patients being female. In the study group, the distribution of the PPIs was given in the following way: 51 dexlansoprazole, 38 lansoprazole, 5 pantoprazole, 5 esomeprazole, and 1 omeprazole. This was done according to the surgeons' preference.

For the control group, the mean scores for pain and swallowing disability were 5.26 and 4.7, respectively, at the 1st postoperative day, 3.9 and 3.12, respectively, during the 1st week, and 2.41 and 1.76, respectively, on the 7th postoperative day. For the study group, the mean scores for pain and swallowing disability were 4.58 and 3.74, respectively, at the 1st postoperative day, 2.57 and 1.87, respectively, during the 1st week, and 1.27 and 0.87, respectively, on the 7th postoperative day (Table 2).

When comparing the study group to the control group, the average pain level was 2.57 and 3.9, respectively, during the first postoperative week (*P* value = 0.001) and 1.27 and 2.41, respectively, at day 7 (*P* value = 0.001). Swallowing disability was also lower in the study group when compared to the control group, 1.87 and 3.12, respectively, during the first postoperative week (*P* value = 0.001) and 0.87 and 1.76, respectively, at day 7 (*P* value = 0.007).

## 4. Discussion

Patients who undergo thyroid surgery often complain of pain and swallowing discomfort [2]. These symptoms are postulated to arise from the endotracheal intubation as well as other factors [6–8]. To our knowledge, there has not been a clear etiology to explain this group of patients' complaints. Recently, researchers have started to look in depth at these symptoms. Patients often complain of a burning throat discomfort/pain, swallowing disability, and throat clearing [3], which mimic symptoms of LPR. LPR has been linked in the past with other aerodigestive tract phenomena, for example, obstructive sleep apnea (OSA) [9]. A correlation between the presence of laryngeal inflammation and the decrease in laryngeal sensation has also been shown [9].

This pilot study looks at patients who are taking PPI in the postoperative period and assesses symptoms of swallowing disability and pain after thyroidectomy. The aim was to determine whether PPIs reduced these complaints. The average pain level for the control group over the course of the 7 days postoperatively was 3.86, which is a moderate pain level. The worst pain was experienced on the 1st postoperative day, where the average pain score was 5.26. At the 7th postoperative day the pain was mild at 2.41. This is consistent with previously published studies from our center that found the pain level after thyroidectomy to be on the milder side of the scale [1].

When comparing these results with the study group, it shows that the study group had an overall significantly milder pain score of 2.8 (*P* value = 0.002). The average pain level on the 1st postoperative day was the highest with a score of 4.58. The study group seems to recover more quickly than the control group with pain scores during the first postoperative week and at the 7th postoperative day lower than the control group at 2.57 and 1.27, respectively (*P* values = 0.001, 0.001). Swallowing disability showed similar results. 

It is interesting to note that when reviewing the data, the groups did not have any significant difference in their initial pain or swallowing symptoms (*P* values = 0.4, 0.12, resp.) suggesting that both groups have had similar initial insult. During the first week patients who were taking PPIs showed significant improvement in their pain symptoms and a quicker recovery of their swallowing ability. This continues until the end of the first week where the study group still showed better symptomatic control.

Recent studies have also looked into laryngeal and reflux symptoms both preoperatively and postoperatively [10, 11]. It has been shown that LPR may be playing a role in nontoxic nodular goiter [11]. Another study has looked into the fact that some patients have swallowing disability despite the absence of a recurrent laryngeal nerve injury [10]. These studies have pointed to the possibility of LPR playing a role in patients postthyroidectomy symptoms [10, 11]. In a recent study, Dekens et al. [12] argues that LPR plays a role in patients' symptoms before and after surgery. Their study suggests that patients who were found to have LPR preoperatively had more symptoms postoperatively. PPIs were suggested as a solution for these symptoms.

 In comparison to other studies, our study focuses on PPI as a treatment for patients' complaints, which are similar to that of LPR. The average pain and swallowing scores over the course of the postoperative period for the control group were 2.15 and 1.96, respectively, compared to significantly better controlled symptoms in the study group with scores of 1.84 and 1.65 (*P* values = 0.001, 0.002). This provides evidence that PPI therapy improved the symptoms of patients in the early postoperative period. This fact reinforces the hypothesis that the insult of a thyroid surgery on the larynx leads to laryngeal inflammation and possibly LPR. Moreover, this laryngeal insult may in fact result from tracheal stimulation during thyroid surgery. Since this group of patients are not paralyzed during thyroidectomy, the tracheal manipulation triggers the cough reflex multiple times throughout the surgery [12].

There are several limitations to this study. There is a selection bias, since only one of the 4 surgeons was using PPIs. Second, the duration, extent, and complications associated with the surgery were not taken into account. Finally, the selection of PPI was not random. 

 To our knowledge this is the first study that studies the effect of PPIs on laryngeal symptoms during the early recovery period after thyroidectomy. A randomized blinded study is warranted to compare the true effects of PPIs on postthyroidectomy pain and swallowing.

## 5. Conclusion

1. The pain and swallowing discomfort suffered by patients following thyroid surgery may be reduced by PPIs given postoperatively.
2. An association between surgical insult following thyroid surgery and LPR needs further investigation.
3. Patients treated with PPIs had less pain and swallowing disability in the first week following thyroid surgery, when compared to patients not treated with PPIs.

## Figures and Tables

**Table:** (a) Please rate the level of throat pain (not the surgery site pain) you had after your surgery

Day	Pain score										
1st day after surgery	0	1	2	3	4	5	6	7	8	9	10
During the first week after surgery	0	1	2	3	4	5	6	7	8	9	10
At day 7 after surgery	0	1	2	3	4	5	6	7	8	9	10

0 = no pain.

10 = worst possible pain.

**Table:** (b) Please rate the level of swallowing disability you had after your surgery

Day	Swallowing disability score										
1st day after surgery	0	1	2	3	4	5	6	7	8	9	10
During the first week after surgery	0	1	2	3	4	5	6	7	8	9	10
At day 7 after surgery	0	1	2	3	4	5	6	7	8	9	10

0 = no swallowing disability.

10 = unable to swallow.

**Table 2:** This table illustrates the mean scores for both groups and their statistical significance during the first postoperative day (POD1), midweek, POD7, the overall average, and the overall average excluding the POD1 (POD2 to POD7).

	Control	Study	Significance
POD1 pain	5.26	4.58	0.4
POD1 dysphagia	4.7	3.74	0.112
Midweek pain	3.9	2.57	0.001
Midweek dysphagia	3.12	1.87	0.001
POD7 pain	2.41	1.27	0.001
POD7 dysphagia	1.76	0.87	0.007
Pain average	3.86	2.8	0.002
Dysphagia average	3.2	2.16	0.005
Pain average excl POD1	2.15	1.84	0.001
Dysphagia average excl POD1	1.96	1.65	0.002

## References

[1] Kalmovich LM, Cote V, Sands N, Black M, Payne R, Hier M (2010). Thyroidectomy: exactly how painful is it?. *Journal of Otolaryngology*.

[2] Lombardi CP, Raffaelli M, De Crea C (2009). Long-term outcome of functional post-thyroidectomy voice and swallowing symptoms. *Surgery*.

[3] Lombardi CP, Raffaelli M, D’Alatri L (2008). Video-assisted thyroidectomy significantly reduces the risk of early postthyroidectomy voice and swallowing symptoms. *World Journal of Surgery*.

[4] Koufman JA, Aviv JE, Casiano RR, Shaw GY (2002). Laryngopharyngeal reflux: position statement of the committee on speech, voice, and swallowing disorders of the American Academy of Otolaryngology-Head and Neck Surgery. *Otolaryngology*.

[5] Reichel O, Dressel H, Wiederänders K, Issing WJ (2008). Double-blind, placebo-controlled trial with esomeprazole for symptoms and signs associated with laryngopharyngeal reflux. *Otolaryngology*.

[6] Pereira JA, Girvent M, Sancho JJ, Parada C, Sitges-Serra A (2003). Prevalence of long-term upper aero-digestive symptoms after uncomplicated bilateral thyroidectomy. *Surgery*.

[7] Sinagra DL, Montesinos MR, Tacchi VA (2004). Voice changes after thyroidectomy without recurrent laryngeal nerve injury. *Journal of the American College of Surgeons*.

[8] McIvor NP, Flint DJ, Gillibrand J, Morton RP (2000). Thyroid surgery and voice-related outcomes. *Australian and New Zealand Journal of Surgery*.

[9] Payne RJ, Kost KM, Frenkiel S (2006). Laryngeal inflammation assessed using the reflux finding score in obstructive sleep apnea. *Otolaryngology*.

[10] Lombardi CP, Raffaelli M, D’Alatri L (2006). Voice and swallowing changes after thyroidectomy in patients without inferior laryngeal nerve injuries. *Surgery*.

[11] Fiorentino E, Cipolla C, Graceffa G (2011). Local neck symptoms before and after thyroidectomy: a possible correlation with reflux laryngopharyngitis. *European Archives of Oto-Rhino-Laryngology*.

[12] Dekens JL, Mastboom WJ, Bultstra G, Oostveen E, Rasker JJ (1999). Coughing reflex induced by electrostimulation of the trachea: a pilot study. *The Lancet*.

